# Prospective randomized trial comparing efficacy and safety of intravenous and intra-articular tranexamic acid in total knee arthroplasty

**DOI:** 10.1186/s43019-020-00079-8

**Published:** 2020-11-18

**Authors:** Moses Man-Lung Li, Jojo Yan-Yan Kwok, Kwong-Yin Chung, Kin-Wing Cheung, Kwok-Hing Chiu, Wai-Wang Chau, Kevin Ki-Wai Ho

**Affiliations:** 1 Department of Orthopaedics and Traumatology, Prince of Wales Hospital, Shatin, Hong Kong, SAR China; 2 School of Nursing, LKS Faculty of Medicine, The University of Hong Kong, Hong Kong, SAR China; 3 Private Practice, Hong Kong, SAR China; 4 Department of Orthopaedics and Traumatology, The Chinese University of Hong Kong, Prince of Wales Hospital, Shatin, Hong Kong, SAR China

**Keywords:** Knee arthroplasty, Tranexamic acid, Randomized controlled trial

## Abstract

**Background:**

Total knee arthroplasty (TKA) is associated with significant perioperative blood loss and postoperative allogenic blood transfusion. Tranexamic acid (TXA) reversibly blocks lysine binding sites on plasminogen molecules and inhibits plasmin formation. Comparisons of the efficacy and safety of intra-articular and intravenous TXA in primary TKA have not previously been reported.

**Methods:**

A prospective randomized trial was conducted in 150 patients who underwent TKA, and these patients were randomized into three groups. Patients in Group A were injected by intra-articular TXA according to body weight (20 mg/kg). Patients in Group B received a standard dose of intra-articular TXA (2000 mg), and those in Group C were infused with TXA according to body weight (20 mg/kg) before tourniquet deflation and again 3 h later. Baseline characteristics and data collected at blood transfusion were compared. Differences among four time points (baseline, day 0, day 2, and day 5) were carried out using ANOVA.

**Results:**

The hemoglobin levels at postoperative day 5 were 10.6 g/dL for Group A, 10.6 g/dL for Group B, and 10.7 g/dL for Group C. The drain output was 399 ml for Group A, 314 ml for Group B, and 305 ml for Group C (*p* = 0.03). Group C had significantly less drain output than Group A after post hoc comparisons (*p* = 0.05), whereas no significant difference was observed between Group A and B (*p* = 0.09) or between Group B and C.

**Conclusion:**

The weight-adjusted dose of TXA administered intravenously significantly reduced the drain output but not the total blood loss when compared with the weight-adjusted dose of TXA administered intra-articularly. No significant difference was observed in the other parameters among the three groups.

**Trial registration:**

The Joint CUHK-NTEC CREC, CRE-2013.644-T. Registered 1 March 2014.

## Background

Total knee arthroplasty (TKA) is associated with significant perioperative blood loss, and frequently, allogenic blood transfusion is required. Such transfusion may lead to infection, delayed recovery, longer hospital stays, and increased mortality, while also increasing the costs [1]. Various methods have been employed and studied to address the problem of blood loss, with tranexamic acid as a widely studied agent.

Tranexamic acid (TXA), a synthetic derivative of the amino acid lysine, is an antifibrinolytic agent that acts by reversibly blocking lysine binding sites on plasminogen and plasmin molecules, which then renders the plasmin unable to bind or degrade fibrin, thus preserving the fibrin clot and leading to hemostasis.

Growing evidence suggests that TXA is effective in reducing perioperative blood loss, hemoglobin drop, and transfusion rates in primary TKA [2]. However, no clear guidelines exist on the best routes and dosages for the administration of TXA. Different routes of administration of TXA, including oral, intramuscular, intra-articular, intravenous, and topical, have been studied [3–5]. To review the efficacy and safety of intra-articular versus intravenous TXA in primary TKA, we conducted a randomized study in a local Chinese population to compare intra-articular and intravenous methods. The aim of this study is to compare the efficacy and safety of intra-articular and intravenous TXA in primary TKA.

## Methods

This is a single-institution, prospective, randomized trial conducted in a tertiary hospital in Hong Kong. Ethics approval was obtained from the Joint Chinese University of Hong Kong–New Territories East Cluster Clinical Research Ethics Committee (The Joint CUHK-NTEC CREC) (Ethics approval number: CRE-2013.644-T). Informed consent was obtained from each subject. This study was in compliance with ICH-GCP and the Declaration of Helsinki. Inclusion criteria were all patients who were diagnosed with primary osteoarthritis of the knee and scheduled for elective primary TKA. Exclusion criteria included known allergy to TXA, diagnoses other than primary osteoarthritis, history of thromboembolic events (deep vein thrombosis, pulmonary embolism, ischemic stroke, and myocardial infarction), anticoagulant intake, and renal impairment with plasma creatinine level more than 200 μmol/l. Patients with preoperative hemoglobin levels of less than 12 g/dL were also excluded as low preoperative hemoglobin level is a known risk factor for postoperative transfusion. TKAs done with navigation guidance or patient-specific instrumentation were excluded to reduce possible confounding factors, as opening of the femoral intramedullary canal can also act as a source of bleeding.

Randomization adopted random numbers generated by a computer. A dedicated experienced research nurse helped to collect the data. Missing data were minimized because of close monitoring by the research nurse. No crossover happened among the three groups.

All patients underwent primary TKAs with general anesthesia or spinal anesthesia. A tourniquet was inflated at the start of the operation prior to skin incision. Medial parapatellar arthrotomy was employed. An intramedullary femoral guide and an extramedullary tibial guide were used. The femoral intramedullary canal was plugged with autologous bone before prosthesis implantation. Cemented posterior stabilized prostheses were implanted in all patients. The tourniquet was then released after the cement had hardened. Closure of the wound followed once hemostasis was achieved. One suction drain was inserted and clamped for 1 hour at the end of the operation. Mechanical prophylaxis for deep vein thrombosis with a sequential compression device was provided postoperatively until the patient began to ambulate.

From March 2014 to February 2016, 256 patients underwent primary TKA, of which 106 patients were excluded because they met one or more than one of the exclusion criteria. At the end, 150 patients entered the study, and they were randomized into three groups. In the first group, intra-articular TXA according to patient’s body weight (20 mg/kg) was infused via the drain after wound closure (Group A (IA 20 mg/kg)). In the second group, a standard dose of intra-articular TXA (2000 mg) was infused via the drain after wound closure (Group B (IA 2 g)). In these two groups, the drains were clamped for an hour after TXA was given and then released. In the third group, intravenous TXA according to patient’s body weight (20 mg/kg) was infused before deflation of the tourniquet, followed by the same dosage 3 h later (Group C (IV 20 mg/kg)).

Baseline demographics including age, sex, height, weight, and body mass index, were recorded. The preoperative hemoglobin and the postoperative hemoglobin levels at day 0, day 2, and day 5 were recorded. The drain output was recorded. The total blood loss was estimated by measuring the change in hemoglobin balance. Previous studies have suggested that, in abdominal and orthopedic surgeries, the blood volume does not change significantly from the preoperative value [6]. Thus, the blood volume (in ml) on postoperative day 5 was assumed to be the same as the preoperative level. The blood volume will be estimated by the Nadler method, based on sex, height, and weight [7]. The loss of hemoglobin was estimated by using the formula described by Lisander [8].

Transfusion triggers included hemoglobin level less than 8 g/dL, development of symptomatic anemia, or urine output less than 0.5 ml/kg/hour for three consecutive hours not responsive to fluid replacement therapy. Postoperative symptomatic deep vein thrombosis was assessed in terms of calf pain, calf swelling, postoperative persistent fever, or persistent tachycardia and was confirmed with ultrasound. All cases and assessments were done and reviewed by specialists from the local joint reconstruction team.

### Statistical analysis

Baseline demographics were summarized using mean standard deviation and range (minimum-maximum) where appropriate. One-way ANOVA and Fisher’s exact test were used to determine the difference in covariates in baseline characteristics, data collected at blood transfusion, and blood test results among the four data collection time points (baseline, day 0, day 2, and day 5 after TKA). Post hoc Bonferroni multiple adjustments were followed to look for significances under multiple comparisons. All statistical analyses were carried out using IBM SPSS version 25 (Armonk, NY: IBM Corp). A two-sided *p* value ≤ 0.05 was considered statistically significant.

## Results

No statistically significant difference was observed in the baseline demographics between the three groups, including age, sex, body weight, body height, and body mass index (Table 1). Clinical examinations were carried out before surgery and no differences were found in diagnosis and lower limb vascular status. Patients using any concomitant antiplatelet or anticoagulant agent were excluded from this study. Moreover, no statistical difference was observed in the complexity, lateral release, synovectomy, or operative time (mean ± SD (Range): Group A = 71.30 ± 11.90 (53–110); Group B = 69.30 ± 9.90 (47–90); Group C = 70.70 ± 11.00 (50–95)).

**Table 1:** Baseline demographics of the patients and information on blood transfusions

Covariates	Group A (IA 20 mg/kg) (*N* = 50)	Group B (IA 2 g/kg) (*N* = 50)	Group C (IV 20 mg/kg) (*N* = 50)	*P* value^#^
Age	67.40 (50–79)	65.82 (54–83)	66.58 (50–81)	0.40
Sex (male:female)	13:37	7:43	14:36	0.22
Side (left:right)	26:24	23:27	26:24	0.86
Height (cm)	154.97 (145–174)	154.44 (138–172)	156.02 (142–171)	0.89
Weight (kg)	68.20 (53–98)	67.76 (54–95)	68.13 (48–99)	0.96
BMI (kg/m^2^)	28.42 (21–40)	28.36 (22–37)	27.99 (20–42)	0.83
ASA	3:37:9	10:34:4	8:37:4	0.15
Length of stay (days)	8.88 (4–34)	7.96 (5–15)	8.70 (5–45)	0.53
Baseline hemoglobin (g/dL)	13.73 (12–16)	13.39 (12–15)	13.41 (12–17)	0.17
Day 0 hemoglobin (g/dL)	12.27 (10–15)	12.06 (10–14)	12.17 (10–15)	0.78
Day 2 hemoglobin (g/dL)	10.60 (8–15)	10.61 (9–14)	10.73 (9–13)	0.74
Day 5 hemoglobin (g/dL)	10.59 (8–13)	10.57 (9–13)	10.69 (8–13)	0.76
Baseline platelet	236.62 (132–378)	225.50 (129–335)	241.68 (137–379)	0.34
Day 0 platelet	212.28 (108–348)	201.28 (111–314)	218.34 (121–339)	0.20
Day 2 platelet	184.04 (109–303)	173.42 (103–291)	193.22 (110–397)	0.11
Day 5 platelet	275.64 (155–458)	263.76 (144–408)	282.62 (162–519)	0.34
Baseline hematocrit	0.41 (0.36–0.48)	0.40 (0.36–0.46)	0.41 (0.36–0.50)	0.90
Day 0 hematocrit	0.37 (0.31–0.45)	0.36 (0.30–0.43)	0.37 (0.32–0.46)	0.92
Day 2 hematocrit	0.30 (0.30–0.40)	0.32 (0.26–0.41)	0.33 (0.28–0.40)	0.09
Day 5 hematocrit	0.30 (0.20–0.40)	0.32 (0.26–0.38)	0.32 (0.24–0.39)	0.22
Drain output (ml)	398.80 (100–1000)	313.80 (70–990)	304.60 (50–1070)	0.03
Calculated blood loss (ml)	886.70 (332–1662)	809.21 (437–1944)	790.01 (251–1536)	0.24
Calculated + unadjusted blood loss (ml)	918.60 (332–1662)	810.74 (437–1944)	790.01 (251–1536)	0.07
Transfusion	4	0	1	0.13
Doppler ultrasound	8	6	2	0.23
Deep vein thrombosis	1	2	0	0.77
Complication	–	Acute coronary syndrome, early infection	Hematoma evacuation	

Keys:

*IA* Intra-arterial

*IV* Intra-venous

*ASA* American Society of Anesthesiologists

*BMI* Bone mineral density

#: *P* values calculated using one-way ANOVA

The preoperative hemoglobin level was 13.7 g/dL for Group A, 13.4 g/dL for Group B, and 13.4 g/dL for Group C (*p* = 0.17). The hemoglobin levels at postoperative day 5 were 10.6 g/dL for Group A, 10.6 g/dL for Group B, and 10.7 g/dL for Group C (*p* = 0.76). The drain output was 399 ml for Group A, 314 ml for Group B, and 305 ml for Group C (*p* = 0.03). Group C had significantly less drain output than Group A in post hoc analysis (*p* = 0.05), whereas no significant difference was observed between Group A and B (*p* = 0.09) or between Group B and C (*p* = 1.00). The estimated blood loss by Lisander’s formula was 919 ml for Group A, 811 ml for Group B, and 790 ml for Group C (*p* = 0.07). Four patients required transfusion in Group A, one patient required transfusion in Group C, and no patient required transfusion in Group B (*p* = 0.13).

For the thromboembolic complication, one patient suffered from deep vein thrombosis in Group A, two patients suffered from deep vein thrombosis in Group B, and no patients experienced deep vein thrombosis in Group C (*p* = 0.77). No pulmonary embolism was observed in any patients. One patient suffered from acute coronary syndrome in Group B. Another patient in Group B suffered from early infection, which required washout. One patient in Group C required arthrotomy and evacuation of a hematoma. No statistically significant differences were observed in the above complication among the three groups (Tables 1 and 2).

**Table 2:** Surgical information on the three groups

Surgical information	Group A (IA 20 mg/kg)	Group B (IA 2 g)	Group C (IV 20 mg/kg)	*P* value
Drain output	398.80 (100–1000)	313.80 (70–990)	304.60 (50–1070)	0.03^a^
Calculated blood loss	886.67 (332–1662)	809.21 (437–1944)	790.01 (251–1536)	0.24
Transfusion needed	4	0	1	0.13
DVT	1	2	0	0.77

^a^ Statistical significance found using one-way ANOVA

*DVT* Deep vein thrombosis

## Discussion

As evidenced by multiple studies, TXA is known to be beneficial in reducing the blood loss and transfusion requirements associated with primary TKA [4, 9, 10]. Various routes of administration of TXA exist, including oral, intramuscular, intra-articular, intravenous, and even topical [11]. For intravenous injection, the plasma peak level of TXA is usually reached in 5–15 min. To suppress fibrinolysis in tissue, a therapeutic plasma concentration of 10 ng/ml and 80% reduction in activity of plasminogen activator should be achieved. Thus, an intravenous dose of TXA of 10 mg/kg is required [12–14]. However, numerous articles report on the dosage of intravenous TXA required. One of the systematic reviews showed a large variation, which ranged from 10 mg/kg to 20 mg/kg, whereas large variations were also reported in the repetition and timing of subsequent doses [15]. Although 10 mg/kg was the more commonly preferred dosage, follow-up dosages of 1-10 mg/kg over 4–30 h were also reported. To simplify administration to a single infusion, a 20 mg/kg regimen would be used [16]. Although using a single infusion of 20 mg/kg may seem to be convenient, the subject’s body weight must still be obtained for calculation of the exact dose. Studies have again found that a single fixed dose has proven to be just as effective as a weight-adjusted dose [17]. In our study design, we forfeited the choice of having a control group that did not use TXA because studies have already shown the benefit of TXA, and providing such a control would have been unethical.

In our center, our statistics show no difference in the total blood loss, hemoglobin drop, or need for transfusion. That is, no definite results indicate whether topical or systemic administration is more beneficial in terms of decreased blood loss, hemoglobin drop, or need for transfusion. In another meta-analysis, two studies report on direct comparisons between topical and systemic administration. The results were inconclusive, with one study stating topical administration was superior in reducing blood loss, while the other study showed no difference [10].

As for the comparison of various routes of administration of TXA, the most commonly compared would be the safety and efficacy between intravenous and intra-articular routes. Previous meta-analyses showed no increased risk of thromboembolic events when intravenous TXA was administered during arthroplasty compared with intra-articular administration [10]. This finding concurs with our data, which showed no difference in thromboembolic events between the intra-articular and intravenous group. However, such events may lead to increased systemic complications. Also, drug activity possibly would be maximized if given intra-articularly, as the site of administration directly affects the activity [2].

In this study, no difference was observed among the three groups, concurring with previous studies, regarding the weight-adjusted vs fixed dose or the route of administration, i.e., intra-articular vs intravenous, in terms of blood loss and hemoglobin drop.

A decrease was noted in the total drain output in the intravenous group compared to the weight-adjusted group. However, the total drain output is only a bedside indicator of discharge from the wound, whereas the hemoglobin drop and the total blood loss would be a more genuine indicator of hemostasis. This difference in output may be attributable to the TXA that had been injected into the joint.

With safety and efficacy bearing similar outcomes in all three groups, the option to choose a preferable way may lie with the cost and efficacy. A single injection is always preferred to repeated injections because it saves manpower and cost. Also, the surgeon would exercise more autonomy as the injection usually would be given intraoperatively, and no need would exist for a second dose if the first dose were administered by intra-articular injection.

A fixed dose may also be more user-friendly compared to a weight-adjusted dose. Thus, the future regimen may lie in a single, fixed-dose, intra-articular administration.

### Limitations of this study

Current evidence suggests many different methods for the administration of tranexamic acid and a variable dosage. Our current study adds to the already large amount of evidence. However, our study, the first such study conducted in Hong Kong, had a simple regimen and few complications. The small sample size may obscure the potential positive results in the group. Ideally, recruitment of a larger sample size would be preferable. Estimation of the blood volume by calculation is another variable factor that may be a limitation. Measuring the actual blood volume, however, is unrealistic. By using Nadler’s formula and calculating all patient blood volumes with the same formula, we believe, however, that the margin of error has been minimized.

## Conclusions

No significant difference was observed in the total volume of blood loss, hemoglobin drop, or thromboembolic events across the three groups. The weight-adjusted dose of TXA administered intra-venously significantly reduces the drain output but not the total blood loss when compared with the weight-adjusted dose of TXA administered intra-articularly.

## Data Availability

The datasets used and/or analyzed during the current study are available from the corresponding author on reasonable request.
